# Risk factors for dislocation after bipolar hemiarthroplasty: a retrospective case–control study of patients with CT data

**DOI:** 10.1007/s00590-020-02819-8

**Published:** 2020-10-23

**Authors:** Tilman Graulich, Pascal Graeff, Ashish Jaiman, Stine Nicolaides, Tarek Omar Pacha, Marcus Örgel, Christian Macke, Mohamed Omar, Christian Krettek, Emmanouil Liodakis

**Affiliations:** 1 Trauma Department, Hannover Medical School, Carl-Neuberg-Straße 1, 30625 Hannover, Germany; 2 Vardhman Mahavir Medical College and Safdarjung Hospital, New Delhi, India

**Keywords:** Hemiarthroplasty, Hip dislocation, Hip arthroplasty, Femoral neck fracture, Posterior acetabular sector angle

## Abstract

**Purpose:**

Bipolar hemiarthroplasty has been shown to have a lower rate of dislocation than total hip arthroplasty. However, as the influencing risk factors for bipolar hemiarthroplasty dislocation remain unclear, we aimed to analyse patient and surgeon-specific influencing risk factors for bipolar hemiarthroplasty dislocation.

**Methods:**

We retrospectively analysed patients who were operated between 2012 and 2018 and had dislocated bipolar hemiarthroplasty and matched them to patients without a dislocated bipolar hemiarthroplasty, operated between 2018 and 2019. The study was limited to patients who received either a pre- or postoperative pelvic computed tomography. Besides demographic, morphologic, and physiologic data, we analysed duration of surgery; ASA score; Charlson Comorbidity Index; Almelo Hip Fracture Score; Parker Score; and acetabular morphology angles including acetabular anteversion angle, posterior acetabular sector angle, posterior wall angle, and acetabular roofing.

**Results:**

We included nine patients with a dislocated bipolar hemiarthroplasty and 30 with a non-dislocated bipolar hemiarthroplasty. Patient-specific factors prompting a higher risk for dislocated bipolar hemiarthroplasty were longer duration of surgery (min) (115 ± 50 vs. 80 ± 27, *p* = 0.01); dementia (56% vs. 13%, *p* < 0.01); smaller posterior acetabular sector angle (°) (96 ± 6 vs. 109 ± 10, *p* < 0.01); and smaller posterior wall angle (°) (67 ± 6 vs. 77 ± 10, *p* = 0.02).

**Conclusion:**

Dementia and insufficient posterior wall angle were associated with higher risk of dislocation in bipolar hemiarthroplasty

## Introduction

Classical indications for bipolar hemiarthroplasty (BHA) are displaced femoral neck fractures (Garden types III and IV) in elderly low-demand patients [1]. When measured using the Harris Hip Score, functional results of HA are inferior as compared to total hip arthroplasty (THA) [2]. However, it has also been reported that with BHA, dislocation rates are significantly lower during the first postoperative year, as bipolar cups are larger and have increased jump distance [3]. Nevertheless, dislocations are still a major concern [1]. Hemiarthroplasty is usually recommended for patients > 80 years or those that have a predicted life expectancy of  < 4 years [1, 4]. Dislocation after THA has been analysed thoroughly and is described to have an incidence of 0.5–3% [5]. The underlying causes of dislocation after THA have been classified by Wera et al. [4] into the following six categories: (1) acetabular component malpositioning, (2) femoral component malpositioning, (3) abductor deficiency, (4) impingement, (5) poly wear, and (6) unrecognized aetiology (idiopathic). Instability has been reported as the most common reason for revision surgery after THA in the USA [6]. Factors associated with higher dislocation rates post-THA are small head size; femoral component malpositioning; cup inclination outside Lewinnek`s safe zone (30°–50°); inappropriate anteversion; unrepaired joint capsule; low-volume surgeon; and patient-specific factors such as neurological deficits (dementia, Parkinson disease), high ASA score, history of spinal fusion, abductor deficiency, body mass index > 35 kg/m^2^, and preoperative Harris Hip Score < 41 [5, 7–11]. Despite lower dislocation rates, BHA is associated with higher periprosthetic fracture rates most likely because of lower bone quality [1, 3, 4, 8, 12–14]. Owing to higher patients’ satisfaction with THA than HA in case of displaced femoral neck fractures, the former is recommended in patients aged < 80 years and with a life expectancy of > 4 years. In patients aged > 80 years, both methods show equal results [1, 25, 4]. Age < 80 years is associated with higher dislocation rates than age > 80 years after HA [4].

Dislocation after BHA seems to be independent from the surgical approach [15]. A recent study has reported that posterior wall acetabular morphology may influence hip stability [16].

Therefore, we aimed to comprehensively describe risk factors for BHA dislocation and attempted to answer the following questions:

1. Are neurological deficits and a higher comorbidity index (i.e. ASA score) associated with higher dislocation rates?
2. Does a larger femoral head size reduce the risk of dislocation?
3. Is posterior acetabular wall morphology an independent risk factor for dislocation?
4. Are there any other factors associated with high dislocation rates?

## Methods

We retrospectively analysed all patients with BHA dislocation that were operated in our institution for femoral neck fracture between 2012 and 2019. The inclusion criteria were posterior dislocated BHA and pelvic CT results. Patients who did not undergo a CT scan were excluded from the study. One patient who was < 18 years and operated for tumour resection was also excluded. Finally, 9 patients with BHA dislocation met our inclusion criteria. These patients were matched to 30 patients that did not experience dislocation and who were treated between 2018 and 2019 with matching surgical technique and also had pelvic CT results. All patients were operated by skilled orthopaedic surgeons in the presence of a fellowship-trained orthopaedic trauma surgery consultant. Utmost care was given to capsular closure. A case–control ratio of 1:3 was assumed sufficient for detection of significant differences. All CTs were performed using a Somatom Force (Siemens Healthcare GmbH, Erlangen, Germany). Image slices were 0.6-mm thick. A 3D reconstruction was performed using the Visage 7.1.11 software (Visage Imaging GmbH, Berlin, Germany).

Patient data were collected from our digital patients’ records. Demographic and morphologic data such as age, sex, and BMI were obtained. We also recorded comorbidities including dementia and Parkinson disease, ASA score, and neurologic decline, and determined the Charlson Comorbidity Index (CCI) with the estimated 10-year survival (%), Almelo Hip Fracture Score (AHS) with the predicted risk of early mortality (%) and the Parker score, a score describing mobility in patients after hip fracture. The cup size, surgical approach, and duration of surgery were the intraoperative factors that were noted. We further determined the kind of infection (superficial/deep) and time since surgery in the case of dislocation.

**Fig. 1:**
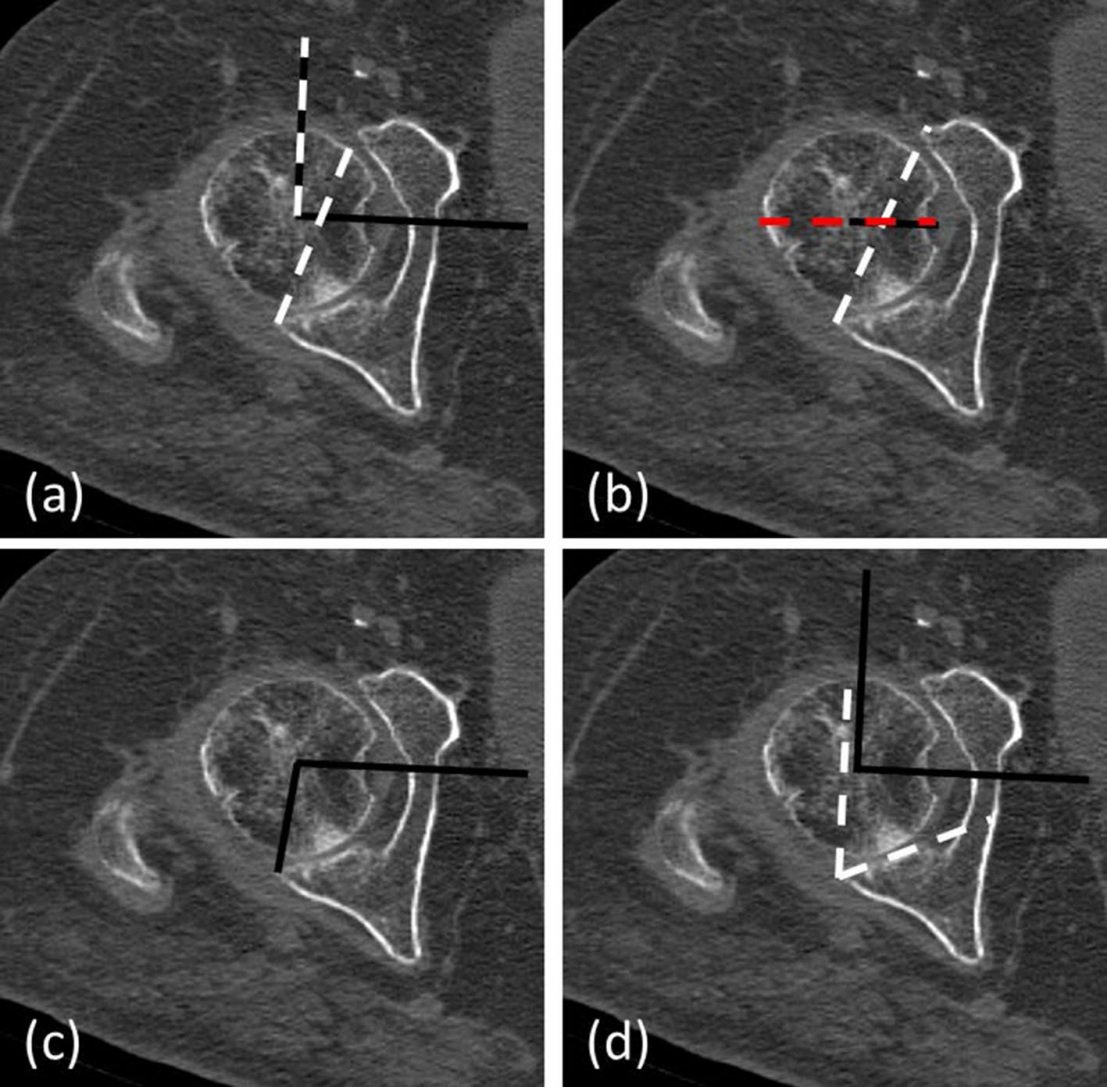
Radiological measurements: **a** in short, an intercapital centre line (ICL) was drawn on true axial images through both femoral heads at the point of the largest diameter in all 3 planes. An orthogonal line to the ICL the ICL90 was drawn. A line between the anterior and posterior acetabular lip of the acetabulum was drawn the anteversion line (AVL). The angle between the ICL90 and AL was measured to determine the AAA. **b** The femoral head coverage was determined using the ICL and the AVL. The part within the acetabulum to the AVL was divided by the whole femoral head diameter. **c** The PASA was determined as described by Valera et al. measuring the angle between the ICL and a line from the femoral head centre to the lateral edge of the posterior wall [17]. **d** The PWA was measured by using the angle between the ICL90 and the tangent to the posterior articular surface area (color figure online)

Polyethylene wear and spino-pelvic imbalance were determined on postoperative radiographs. Radiological evaluation of CT-scans was performed by a trained orthopaedic surgeon (PG), who was blinded to patients’ group allocation. Acetabular anteversion angle (AAA), posterior acetabular sector angle (PASA), posterior wall angle (PWA), and acetabular roofing (%) were evaluated from CT scans. Methodology described by Fullam et al. [15] was used for radiological evaluation. In short, an inter-capital centre line (ICL) was drawn on true axial images through both femoral heads at the point of maximum diameter in all 3 planes. An orthogonal line to the ICL—the ICL90 was drawn. A line between the anterior and posterior acetabular lip of the acetabulum was drawn the anteversion line (AVL). The angle between the ICL90 and AVL was measured to determine the AAA. The PASA was determined as described by Valera et al. [17] i.e. by measuring the angle between the ICL and a line from the femoral head centre to the lateral edge of the posterior wall. The PWA was measured by using the angle between the ICL90 and the tangent to the posterior articular surface area. The femoral head coverage/roofing was determined using the ICL and AVL. The part within the acetabulum to the AVL was divided by the whole femoral head diameter to determine the amount of femoral head coverage (% of total) (Fig. 1).

### Statistical analysis

Data were tested for normal distribution using the Shapiro–Wilk test. For comparative statistics, in case of normal distribution, *t*-test was used. A multivariate ANOVA (MANOVA) was performed. If data were not normally distributed, Mann–Whitney U test was used. Non-normally distributed data are presented as median with interquartile rang, and normally distributed data as mean ± standard deviation. A post hoc power analysis was performed. A *p*-value < 0.05 was considered to indicate statistical significance.

### Institutional review board approval (IRB approval)

**Fig. 2:**
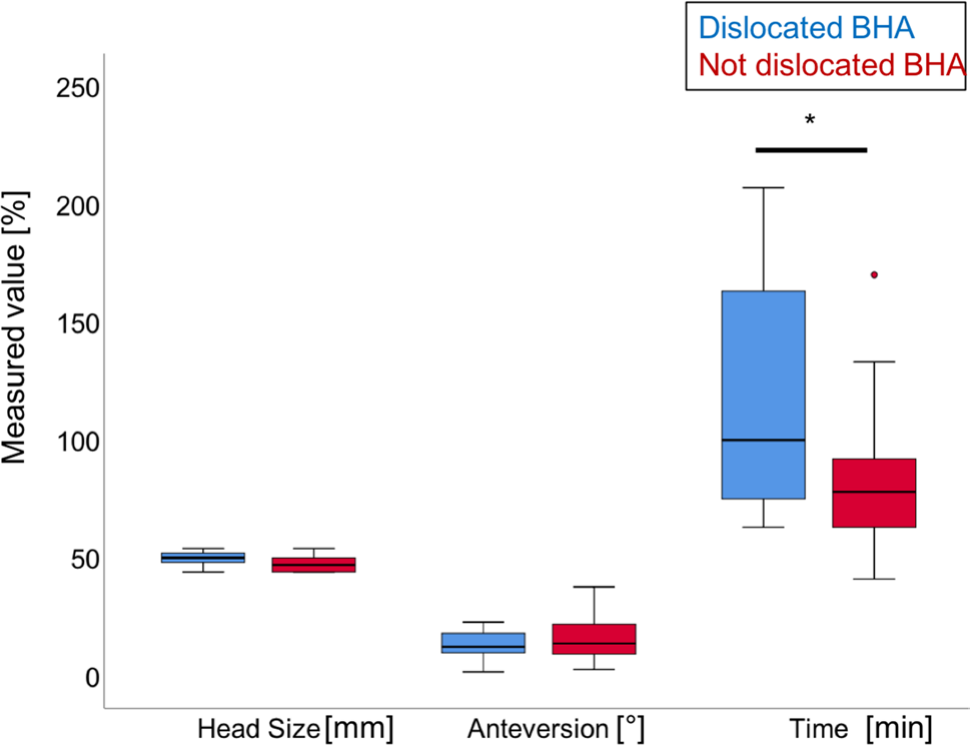
Univariate Analysis. Comparison of operative parameters between dislocated BHA and non-dislocated BHA Blue: Dislocation group, red: Control group, *:*p* < 0.05. Head Size: external head size. Anteversion: femoral shaft anteversion. No differences in femoral shaft anteversion were observed between both groups. Time: operation time. Operation time was longer in patients with dislocated BHA than in patients with non-dislocated BHA (color figure online)

For this study, IRB approval was not required, because the investigator did not obtain any data through interventional interaction and did not present any identifiable personal information (Fig. 2).

## Results

### Demographic, morphologic, and physiologic data

We included 39 patients (mean, 83 years; range, 64–98 years) in the study. The 9 patients with a dislocated BHA (79 ± 7 years) did not show significantly difference in age than the 30 patients without dislocated BHA (84 ± 7 years) (*p* > 0.05). No differences were noted between the BMI of both groups (22 ± 3 vs. 23 ± 5; *p* > 0.05). Sex had no influence on dislocation rates (*p* > 0.05). Dementia with a prevalence of 56% was significantly more common in the dislocation group than the control group (13%) (*p* = 0.009; OR: 7.6). No patient had Parkinson disease. Infection rates, classified as deep infections, were significantly higher in the dislocation group (40 vs. 0%; *p* = 0.01). The average time to first dislocation was 8 months (range: 0–54 months) (Table 1).

**Table 1:** Patient data

	Total (*n* = 39)	Dislocated BHA (*n* = 9)	Non-dislocated BHA (*n* = 30)	*p* value
Sex/male (total/%)	14/35.8	2/22	12/30	0.448
Age (years)	81 ± 13	79 ± 7	84 ± 7	0.139
BMI (kg/m^2^)	23 ± 5	22 ± 4	23 ± 5	0.634
Dementia (total/%)	9/23	5/56	4/13	0.007
Parkinson (total/%)	3/7.6%	0/0	3/10	0.326
Charlson comorbidity index	6 ± 2	6 ± 1	6 ± 2	0.635
Estimated survival	15 ± 22	7 ± 10	17 ± 24	0.240
Almelo hip score	8 ± 3	8 ± 2	8 ± 3	0.815
Almelo predicted risk	5 ± 6	5 ± 4	6 ± 6	0.577
Parker score	6 ± 2	6 ± 3	6 ± 2	0.085

### Comorbidity scores

**Fig. 3:**
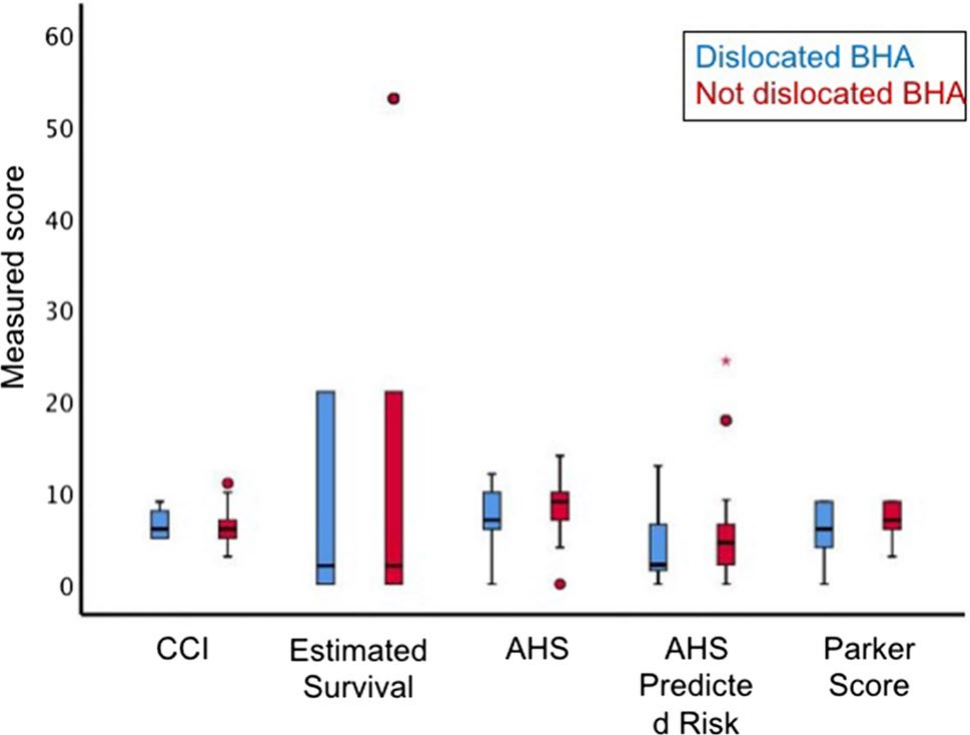
Comorbidities in the dislocation and control group. Blue: dislocated BHA, red: non-dislocated BHA, CCI: Charlson Comorbidity Index, Estimated survival: Estimated survival based on CCI, AHS: Almelo Hip Score. No differences could be observed between both groups (color figure online)

Upon comparing the dislocated BHA with the non-dislocated BHA, the ASA score was 2.4 ± 0.5 versus 2.7 ± 0.5 (*p* = 0.138), CCI was 6.5 ± 1.4 versus 6.2 ± 2.0 (*p* = 0.672), estimated survival was 6.9 ± 9.7 versus 17.4 ± 24.2 (*p* = 0.192). Further, the AHS, Almelo predicted risk, and Parker scores were also not significantly different between both groups (*p* > 0.05) (Table 1 and Fig. 3).

### Radiological measurements

**Fig. 4:**
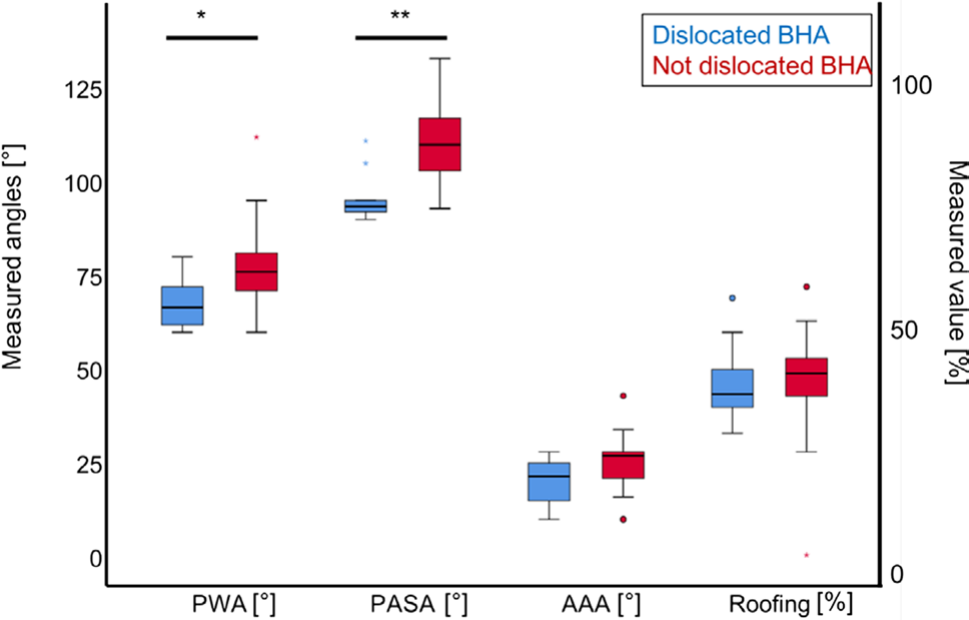
Univariate Analysis. Comparison of acetabular angles and roofing between dislocated BHA and non-dislocated BHA. Blue: dislocated BHA red: non-dislocated BHA, **p < 0.01, *p < 0.05. PWA: posterior wall angle, PASA: posterior acetabular sector angle, AAA: anteversion acetabular angle, Roofing (%): Femoral head coverage by the acetabulum. dislocated BHA shows significantly lower values for PWA, PASA, and AAA but no differences in acetabular roofing compared to non-dislocated BHA

Most importantly, both PASA and PWA were significantly smaller in the dislocated BHA group than in the non-dislocated controls (PASA: 96 ± 6 vs. 109 ± 10; *p* < 0.01; OR: 12.7; power: 99.6%), (PWA: 67 ± 6 vs. 77 ± 10; *p* = 0.02; OR: 7.3; power: 97.9%). The AAA was not significantly smaller in dislocated BHA than non-dislocated controls (21 ± 5 vs. 25 ± 6; *p* = 0.10; power: 90%). The acetabular roofing showed no difference between both groups (Fig. 4 and Table 2). There was no evidence of macroscopic poly wear on radiographs, as the metal head did not show radiographic evidence of eccentricity in the cup. All patients had post-traumatic fracture of the femoral neck without any other systemic musculoskeletal disease, spino-pelvic imbalance, low back pain, or radicular pain.

**Table 2:** Radiological measurements

	Total (*n* = 39)	Dislocated BHA (*n* = 9)	Non-dislocated BHA (*n* = 30)	*p* value
Acetabular roofing (%)	47 ± 12	47 ± 11	47 ± 13	0.939
AAA (°)	24 ± 7	19 ± 5	25 ± 6	0.105
PASA(°)	106 ± 11	96 ± 6	109 ± 10	0.001
PWA (°)	74 ± 10	66 ± 6	77 ± 10	0.02

### Operative technique

Cup size in dislocated BHA was not significantly bigger than in the non-dislocated controls (50 ± 3 vs. 47 ± 3 *p* = 0.051). The duration of surgery was significantly longer in patients with dislocated BHA than in the controls (115 ± 50 vs. 80 ± 27; *p* = 0.01; OR: 4.5; power: 79.5%) (Fig. 2). Surgical approach showed no differences between both groups, with 7 patients (78%) having a lateral approach and two patients (22%) having a posterior approach in the dislocated BHA group than the 19 (63%) lateral versus 11 (37%) posterior approach in the non-dislocated control group (*p* = 0.33) (Chi-square test).

## Discussion

The present study provides comprehensive information about risk factors associated with BHA dislocation. Our findings revealed that dementia is associated with a risk for dislocation. Further, cup size was not associated with higher dislocation risk. Last, posterior acetabular wall morphology with smaller PASA and PWA were associated with higher dislocation rates.

Neurological deficits are known to negatively influence the postoperative outcome after THA [6, 10, 18, 19]. Interestingly, ASA score, CCI, AHS, and Parker Score did not differ significantly between both groups. However, previously published data have shown a high association of comorbidities with the risk of THA dislocation; contradictory to our expectation, we could not observe these differences in our patients after BHA [6, 10, 20]. We believe that the small sample size is the main reason for not detecting significance of comorbidities.

All patients were operated upon by using a 28-mm internal head and different sizes of external cups (44–54 mm). Our results of smaller cup sizes tending to be associated with less dislocation rates after BHA are contradictory to the literature. To our knowledge, the small sample size is the most likely reason for this discrepancy [21].

Furthermore, longer operation time correlated with a higher dislocation risk. We believe that this is related to less experienced surgeons, which has also been reported in literature that low-volume surgeons show higher complication rates in primary THA and BHA than high-volume surgeons [10, 22]. Nevertheless, patients with both dislocated BHA and non-dislocated BHA were operated by multiple surgeons (10 vs. 14 surgeons).

Interestingly, as previously shown in the literature, younger patients who were < 80 years old tended towards higher dislocation rates than those > 80 years [4]. This might be due to the more active lifestyle of younger patients, or because of less bony coverage owing to fewer posterior osteophytes in younger patients.

It has been shown that the bony acetabular coverage measured by a low centre edge (CE) angle has a strong association for a higher risk for dislocation [18]. Recently Kizkapan et al. [23] described a strong correlation of decreased CE angle and, more importantly, a reduced femoral offset to be associated with dislocation in BHA. This shows that although operative technique is an important factor, patient-specific anatomy is an equally important and individual factor that can influence the odds for dislocated BHA.

As for most BHA dislocations and as noted in our study, all nine dislocations were posterior dislocations. We believe that a larger bony posterior wall has a high impact and protective function to prevent posterior BHA dislocations.

Recently, we also described the reference values for the posterior wall coverage with a mean PASA of 100° and PWA of 72° in a healthy patient collective [16]. Our data with reduced PASA and PWA in patients with dislocated BHAs (PASA: 96 ± 6 vs. 109 ± 10; *p* < 0.01) and (PWA: 67 ± 6 vs. 77 ± 10; *p* = 0.02) show that a more open acetabular posterior wall is associated with a higher risk for BHA dislocation.

The debate on influence of surgical approach on dislocation is ongoing. Both the direct lateral approach and posterior approach have been used for BHA, with a tendency towards better functional outcome after the posterior approach; however, it is associated with higher dislocation rates when compared to the lateral approach [15, 24]. In our study, we did not observe significant differences with respect to this aspect of surgery.

As an extrapolation of this study’s data, we devised an in-house algorithm for elderly patients with femoral neck fracture. All patients with a displaced femoral neck fracture (Garden type III or IV) without any clinical sign of hip arthrosis like pre-existing pain should be included and considered as potential candidates for BHA arthroplasty. In the case of dementia, we would either perform a pre-operative CT scan for measurement of acetabular posterior wall indices or subjectively evaluate the posterior wall intraoperatively and label it as closed or open. In the case of a PASA < 95.5° and PWA < 71.5° or an intraoperative open acetabulum, we would consider a dual mobility cup or a THA with a constrained liner, instead of BHA. In the case of no dementia and PASA and PWA > 95.5° and 71.5°, respectively, or an intraoperative closed acetabulum, we would consider a BHA arthroplasty. Alternatively, in place of measuring the posterior wall angles, the decision can be made by 3D reconstructions as recently described [16].

Our study has some limitations. The retrospective nature is the first limitation of this study, as investigators were required to rely on the availability and accuracy of medical records. Second, no subject in the control group had a torsion difference CT, and only 5 patients in the dislocation group had a torsion difference CT. This means that femoral stem anteversion could not be compared between the 2 groups. Last, the sample size was very small. Although for some parameters like radiological measurements, a high post-hoc power (79.5–99.6%) could be achieved, we believe that further parameters like comorbidity scores and neurological deficits (e.g. Parkinson’s disease) are underpowered (power: 5–51.4%) and might not reflect the clinical importance of these parameters.

## Conclusion

To the best of our knowledge, this is the first study to evaluate CT data to detect anatomy-associated risk factors for dislocation after BHA for femoral neck fracture.

While many studies concentrated on dislocation risks for THA, this study comprehensively described risk factors for BHA dislocation showing that besides neurological deficits like dementia, the posterior wall anatomy is an important independent factor associated with dislocation rates.

Considering that dislocation after BHA is not so common, further large-sampled, multi-centre, prospective studies should be conducted to validate our results.
